# On Mental and Physical Life in Relation to Time

**Published:** 1863-07

**Authors:** 


### Art. III.—ON MENTAL AND PHYSICAL LIFE IN RELATION TO TIME.

### Part II.

In treating of psychical life in relation to time,* we started from the scientific unity, consciousness. In treating of physical phenomena, we must start from the scientific unity of the *organism*, and from this point must proceed all our radiations with the science of life.

We saw there is a prolongation of consciousness in time, and from this we have our notions of personal identity. Here we find a prolongation of the organism in time, "organization in action," and it is from this only that we can form any notion of the nature of physical life.

We saw that the unity and continuity of consciousness forms the ultimate fact of psychology. Here the unity and continuity of organization (as regards both structure and function) forms the ultimate fact of psychology. We saw that a synthesis of the ultimate fact of psychology breaks it up into a series of states or mental functions—reason, sensation, and the will, which manifest themselves according to certain laws. Here, on the other hand, a synthesis of the ultimate facts of physiology decomposes it into a series of functions—reproduction, nutrition, and death, which manifest themselves according to certain definite laws, vital and physical.

We found that mental states succeed each other with a rapidity far above human calculation—and here our parallelism ends, for these physical states succeed each other at a rate quite reducible to numerical calculation, and it is the object of this division of our thesis to notice the order of succession of certain physical phenomena.

---

* P. 224.

Having taken the *organism* as our starting-point of investigation, it may be well to understand what is exactly meant by the term. The organism is an aggregation of differentiated parts, so arranged as to form a nicely calculated whole, endowed with vital and physical forces, in virtue of which it expresses all those functions characteristic of life. We need scarcely remark, that this calculated whole, this individuality, the organism lives under the category of time. "The organized being," says Professor Goodsir, "has a stated period of existence, which, however, is not absolute, but varies according to the circumstances in which it exists, the animal dies, and *death* is a process of life." The organism is indeed characterized by the perpetual changes it is continually passing though; the animal is born, it grows, it becomes mature, it declines and dies—the life of the organism is thus not only limited in time, but its duration, whether long or short, is mapped out into a series of stages or epochs, corresponding to its progressive development from the cell to the perfect being, as well as to its retrogression, from the full-grown being down to the molecule and granule—its primary constituent elements. It is a trite remark that all organized beings live under the categories of time and space, the duration of the former and limitation of the latter depending on certain well-defined physical conditions. This law assumes more importance when we leave the individual, the organism itself, and investigate the relations existing between species or genera and the great element, time. To the geologist, especially, the study of such relations has very great interest indeed, for on the correct definition of such depends the true appreciation of the laws of representation and distribution, without which palæontological science would be almost a dead letter. Thus geologists group species together, according to the time of their apparent duration and existence. Each species, whether animal or vegetable, has a duration in time limited, as well as a definite area in space, in other words, each species has a definite area in time and space. The life of the organism depends on certain physical conditions, and as these are favourable or the reverse will its existence be extended or diminished—so geologically is it with the species, for according to its duration in time is its power of enduring various physical conditions. According to the late Edward Forbes, genera, when applied to geological distribution, occupy a definite area in time, the area in many instances being very limited, and their ranges restricted. Indeed, very few genera have ranged through the periods from the first appearance of life to the present time. Of course this restriction of range and limitation of area becomes more apparent as we descend the scale of classification, (if I may so speak,) for many of the varieties, families, and even species, found in some of the earlier rocky formations, are now quite extinct. In the words of the great Hugh Miller, "the Omnipotent Creator never repeats to himself, but breaks, when the parents of a species have been moulded, the die in which they were cast." Thus we may see how important to the naturalist is the study of those relations existing between the element time, and the organism, the species, and the genus. *Time*, to the geologist, is a mighty element, linking the present to all the past; is a key to those volumes of stone in which is portrayed "The unity of the authorship of a wonderfully complicated design, executed on a groundwork broad as time itself, and whose scope and bearing are deep as eternity."

All vital actions or states, as expressed in organization, would seem to succeed each other according to *laws* having a certain definite or fixed relation to *time*. The most apparent of these are two in number—viz.,

(*a*) The law of occurrence (simple sequence).

(*b*) The law of periodicity, or recurrence.

The simple sequence of any vital action (normal or abnormal) is a fact so patent to every observing mind, that it requires no demonstration. We know, for example, that the union of the germ cell and sperm cell is followed by the evolution of a new product; that this product of conception goes through a distinct series of developmental stages, and that the duration of this series of events terminates in a few days, weeks, or months, according to the species of animal in which the evolution takes place. We know that a cutaneous eruption is a phenomenon of variola (for example), and that this eruption is, in orderly succession, evolved, maturated, and desiccated, and that each of these successive stages is accompanied by symptomatic phenomena peculiar to itself—as the primary and secondary fever, the critical discharge, &c., &c. To use another example, we know that before an exudation of liquor sanguinis can be removed from any tissue, it must either rise (by progressive developments) in the scale of cell-life, and be eliminated as pus, or return into its primordial elements, and be absorbed into the blood. Here we see an order of events following each other in the relation of either cause or effect, and each succession having a definite duration, a definite existence in time. A knowledge of the order of succession of vital phenomena, normal and abnormal, constitutes, according to Professor Laycock, the foundation of etiology—the science of the causes of disease. "By removing the cause (of disease) is meant simply interrupting the order of the morbid phenomena—a breaking a link or links in the chain of causation."

It is scarcely necessary to enter into detail as to the causes which regulate the occurrence of vital changes in the organism, yet a passing notice may not be out of place; and in doing so let us follow the three great processes of organic life—viz.,

*a. Reproduction* as expressed in *Development.*

*b. Nutrition* as expressed in *Growth.*

*c. Death* as expressed in *Degeneration.*

The conditions regulating the developments of any primordial germ-cell are of two kinds, inherent and vital; and, secondly, external and physical. "Every germ-cell possesses a germinal capacity in virtue of which it develops itself into a structure of its own specific type." But this germinal capacity is "brought into play by influence external to the cell, and such are a proper nutrient material, a certain temperature, a fit locality, &c. Taking *temperature* as a modifying cause, we find that its variations have great influence over the occurrence of the vital changes in development. The different changes which occur in vegetables, as dependent on the seasons of the year, show well the importance of a proper temperature for the evolution of the young plant." For the germination of certain plants a certain temperature is requisite; and as this occurs at a particular season of the year (spring or summer) the period of germination is thus determined. If this period of germination be delayed by seasonal changes, the evolution of the embryo-plant is retarded. Again, according to Humboldt, there is "upon the earth's surface a geographical distribution of plants, according to its various climates, which form so many zones of vegetation, from the pole to the equator." This law holds good in the animal kingdom. The influence of temperature as a condition of development is not more beautifully illustrated than in what takes place in the preservation of many species of *insects.* In the warmth and sunshine of summer the atmosphere seems perfectly alive with insects of every form, colour, and size; but in the *cold* and sunshine of winter they are not visible. Of the two conditions —heat and light—the former is absent in winter; and this would seem to be the chief cause of the great mortality among these tiny existences. How are they reproduced? for by reproduction alone can the species be extended in time. It would seem that they are for the most part, if not all, oviparous, and their eggs are endowed with the quality of preserving the principle of vitality for several months in circumstances which would have proved fatal to the animal itself. Both Spallanzani and Hunter have tested the degree of cold to which the eggs of insects may be subjected without injury: the latter observer enclosed the eggs of the *moth, silkworm,* &c., in a glass vessel, and buried it five hours in a mixture of rock-salt and ice; and he tells us that though the thermometer fell six below zero, caterpillars came from all the eggs. Professor Simpson made experiments of a like nature, which tended to show that any sudden change of temperature excites the eggs of some insects into rapid evolution. Thus he placed one-half of a nest of insect ova into an ice-house, and allowed them to remain for some months, and upon removing them their evolution into larvæ was almost immediate, whilst the remaining half of the batch (not confined to the ice-house) were not hatched for some time after.

*b.* Of *Nutrition* as expressed in Growth in its relation to *Time.*

While reproduction maintains the life of the species, we find that the individual owes the maintenance of its life to the function of *nutrition.* Each species is a long chain, and each individual a link in that chain. The individual has a limited and unique existence in *time,* which, short as it must be, can be shortened by the influence of unfavourable conditions. We need scarcely say that among the conditions favourable to the life of the individual, the most essential are the due performance of the nutritive operations, by which we mean all those vital and physical actions as comprehended in the idea of cell-growth. The influence the nutritive process has in extending or diminishing the duration of the organism may be seen in the influence it exerts on the comparative duration of its component parts—viz., the different tissues and structures. "Every cell," says Dr. Carpenter, "lives for itself, and by itself, like each of the solitary cells of the humblest protophyte; and if the necessary conditions be furnished, it may continue to live and to grow quite independently of the organism of which it originally formed part." Schwann, in his "Cell Theory," elaborates this same idea, but adds that the cell "is at the same time directed by the influence of the entire organism in such a manner as the design of the whole requires." "As every component part of the most complex organized fabric has an individual life of its own, so must each have a limited duration." This duration, according to Dr. Carpenter, varies greatly in the different kinds of organized tissue; and he has classified the different tissues according to the length of their duration. Thus, in the first class, he places *temporary cells,* or those which have a very transient existence, and which die and disappear without undergoing any higher development. To this class belong all those cells which appear in the formation of the pollen grains and ovule of flowering plants, and in the germinal vesicle of the ovum of animals. Such also are most of the secreting cells, whose allotted functions being all performed within a few days or hours, die as soon as this is the case. In another class the same author places those tissues whose functions are to be indefinitely prolonged, such prolongation being seen only when their formation, instead of being vital, is simply physical; as in the case of parts that afford mechanical support, or resist tension, or supply elasticity. As examples, he mentions the heart wood of trees, the bones and shells of animals, and, still more, their hair, hoofs, horns, &c., which may remain unaltered through an unlimited series of years. In another division we have the component tissues of the nervo-muscular apparatus, the duration of which diminishes according to the degree of their vital energy.

*c. Of Death,* as expressed in Degeneration, as influencing the duration of the organism. As we remarked above, the organized being has a stated period of existence, which is not absolute, but varies according to the circumstances in which it exists. The animal dies, and "death is a process of life." During life there is a continual and energetic struggle between the physical and vital forces, and death is the triumph of the former over the latter. In a physiological sense, degeneration is neither more nor less than the history of old age. But here we must view it in a pathological sense, for it is simply a morbid change in the structure of parts. It would seem to be a law in organic life that every vital action involves a certain amount of waste of tissue. "Every action of the nervous and muscular systems involves the death and decay of a certain amount of the living tissue, as is indicated by the appearance of the product of that decay in excretions." There is, so to speak, a local death constantly going on in the organism, hence the great and constant occasion for the excretory operations. This waste or local death may take place either through vital changes in the tissue itself, or physical or external influences. Many cells, after having performed their allotted function, immediately die and disappear. In most instances, their death is due to what is called "granular degeneration." The physiologist tells us that by this means Nature often accomplishes the removal of redundant structure, thus the fibres of the uterus undergo (after delivery) an entire fatty transformation, so that in the course of time the tissue surrounding the fibres becomes absorbed, and the uterus returns to its normal volume. Again, this process is seen in the shedding or exuviation of the epithelial and epidermic structures which takes place to a great or less extent in all classes of animals. In many morbid processes we find Nature employing this kind of death for the removal of matter obnoxious to the system. Thus, in the process of resolution (of an inflammation) pus-cells are developed from a blastema found in the exudation which, after attaining full development, break down into molecules and granules, in fact, return to a fluid state somewhat resembling the liquor sanguinis, and in this state are absorbed into the system. In such cells, *death* would seem to be as essential a phenomenon as their growth, and it seems always to be regulated by the fulfilment of their function, for we find the moment the pus-cell has attained full development, it breaks down and ceases to exist, so that their duration is in the inverse proportion to their vital activity.

The law of *Periodicity* presents a subject at once interesting and mysterious. Periodicity we understand to be the disposition of certain phenomena to occur at stated periods, these periods being regular and according to distinct series. Vital periodicity would seem to depend on causes *within* as well as without the organism. It would also appear that a mutual relation existed between these two kinds of causes, the internal (vital) resisting the external (physical), and the latter in their turn regulating, to a certain extent, the operation of the former. " Every animal has a time for development and growth, a period of middle age, in which the body strives to maintain an unaltered mass, and an epoch of decrease, which is concluded by natural death." These periodical changes are explained by the mode of arrangement which prevails in the organic creation, and constitute the necessary results of certain stages of growth, and follow inevitably from the previous and accompanying condition. " Since many creatures prepare, during the cold of winter for the approaching heat of spring, and since women menstruate every four weeks during all seasons indifferently," it follows that the periodicity of those functions depends, not absolutely perhaps, but to a great extent, on causes within the organism. The influence of an external cause on periodicity is very apparent in almost every occurring function. Thus the influence of light, as tested by the diurnal revolution, on the production of sleep, the flowering of plants, &c. ; the influence of heat and cold, as tested by the annual revolutions, on the reproductive function of both plants and animals. As remarked above, these two causes are seldom found separately but in mutual relation, one either resisting or being governed by the other. This is beautifully seen in the adaptation which exists between organized beings and the changes of the seasons. "It is evident," says Dr. Duncan, "that some peculiar provision has been made in temperate climates for the preservation of organized existences during winter. In that season they are not in the same condition as in other seasons of the year." " Not only are these peculiar provisions for preserving animal and vegetable life, in our temperate climate, during the cold of winter, but the whole classes of organized beings, which exist in any climate, and adapted to all the ordinary changes of their peculiar locality." Every parallel of latitude is distinguished by its peculiarity of weather, its longer or shorter duration of mildness or of rigour, of rain and drought, of light and darkness; and to all these varieties, plants and animals belonging to that latitude, are adapted. Upon this law Humboldt divides the earth's surface into so many zones of vegetation, each zone indicating the habitat of certain species. The changes in the vegetable kingdom, which begin in autumn and are consummated in winter, do not seem to be brought about by the chilly nature of the season alone, but are the indications of a circle of changes inherent in the plants, which correspond with the cycle of the year. The mutual relation existing between organic and inorganic periodicity, if we may so call meteorological and other recurring phenomena, is not more beautifully illustrated than in the great function of sleep. Setting aside altogether the question as to the physical causes of sleep, it seems manifest, that the great object in view is the reparation of exhausted power. This need extends in the most general sense to life in every form, and the periodicity of this function would seem to depend upon and provide for this necessity. It is well known that those vital changes which, with other external causes, bring about sleep, occur at stated periods, these periods recurring every twenty-four hours, and coinciding with the return of night and day. But why this coincidence with night and day? Simply because those *inner* revolutions of vital changes necessary to produce sleep are to a great extent regulated by *external* revolutions, viz., meteorological changes. Thus the absence of light would seem to have great influence in inducing sleep. The direct influence of light on sleep might be difficult of proof, yet it is rendered at least probable by the effect usually produced on the approach of darkness on animals in general, but more especially on birds, for, with few exceptions, all birds betake themselves to sleep as soon as night approaches, and if darkness should anticipate night by many hours (as by an eclipse of the sun), they still give the same indications of composing themselves to sleep as at the regular period of sunset.*

Again, Dr. Carpenter thinks that the diurnal withdrawal of light produces a diminution of the activity of the vital processes of plants, this diminution occurring in a periodical manner—in other words, the plants sleep. We have proof of this in the fact that artificial light and warmth will cause many flowers and leaves to erect themselves for a time, and by proper management the usual periods may be completely reversed. What is true of sleep is equally so of all vital phenomena

---

* Macnish.

which are periodical in their nature. They may differ among themselves in this, that their *inner* revolutions may follow meteorological changes of different duration, some of a diurnal type (as sleep), others a monthly (as menstruation), others, again, at intervals which are multiples of a month (as the periods of utero-gestation, dentition, &c.), and others, again, seasonal and annual, and multiples of these (as the periods of hibernation). In conclusion, we would remark that the functions of all organized beings which are periodical in their nature suffer, at certain periods, intervals of augmentation and cessation, and that periodicity proceeds partly from the original endowments of their organization, acted on or called into exercise by forces external to itself.

---

